# Obstacles to Health Big Data Utilization Based on the Perceptions and Demands of Health Care Workers in South Korea: Web-Based Survey Study

**DOI:** 10.2196/45913

**Published:** 2023-04-13

**Authors:** Yoon Heui Lee, Yu-Jin Jang, Soo-Kyoung Lee

**Affiliations:** 1 Department of Nursing, Graduate School, Keimyung University Daegu Republic of Korea; 2 Dongsan Hospital, Keimyung University Daegu Republic of Korea; 3 College of Nursing, Keimyung University Daegu Republic of Korea

**Keywords:** demand, health big data, health care worker, obstacles, perception, utilization

## Abstract

**Background:**

This study focuses on the potential of health big data in the South Korean context. Despite huge data reserves and pan-government efforts to increase data use, the utilization is limited to public interest research centered in public institutions that have data. To increase the use of health big data, it is necessary to identify and develop measures to meet the various demands for such data from individuals, private companies, and research institutes.

**Objective:**

The aim of this study was to identify the perceptions of and demands for health big data analysis and use among workers in health care–related occupations and to clarify the obstacles to the use of health big data.

**Methods:**

From May 8 to May 18, 2022, we conducted a web-based survey among 390 health care–related workers in South Korea. We used Fisher exact test and analysis of variance to estimate the differences among occupations. We expressed the analysis results by item in frequency and percentage and expressed the difficulties in analyzing health big data by mean and standard deviation.

**Results:**

The respondents who revealed the need to use health big data in health care work–related fields accounted for 86.4% (337/390); 65.6% (256/390) of the respondents had never used health big data. The lack of awareness about the source of the desired data was the most cited reason for nonuse by 39.6% (153/386) of the respondents. The most cited obstacle to using health big data by the respondents was the difficulty in data integration and expression unit matching, followed by missing value processing and noise removal. Thus, the respondents experienced the greatest difficulty in the data preprocessing stage during the health big data analysis process, regardless of occupation. Approximately 91.8% (358/390) of the participants responded that they were willing to use the system if a system supporting big data analysis was developed. As suggestions for the specific necessary support system, the reporting and provision of appropriate data and expert advice on questions arising during the overall process of big data analysis were mentioned.

**Conclusions:**

Our findings indicate respondents’ high awareness of and demand for health big data. Our findings also reveal the low utilization of health big data and the need to support health care workers in their analysis and use of such data. Hence, we recommend the development of a customized support system that meets the specific requirements of big data analysis by users such as individuals, nongovernmental agencies, and academia. Our study is significant because it identified important but overlooked failure factors. Thus, it is necessary to prepare practical measures to increase the utilization of health big data in the future.

## Introduction

The fourth industrial revolution has ushered in a data economy in which data utilization serves as a catalyst for the development of other industries and creates innovative businesses and services. In the 21st century, data have emerged as the new oil, becoming the key resource in determining the competitiveness of individuals, countries, and companies [1,2]. In this context, it must be noted that the rapid increase in digital data production and the development of analysis technology have stimulated interest in big data analysis and utilization in various fields. This data explosion is also prevalent in the health care industry [3]. In health care, if medical, clinical, genomic, and personal health information are integrated into data and analyzed, it will not only optimize customized health care for individual conditions and the environment [4] but also strengthen self-determination and self-directed health care [5]. Health big data analysis will also significantly influence decision-making in the medical field [6,7].

This study focuses on the potential of health big data in the South Korean context. South Korea has a world-class health big data reserve and information technology infrastructure. The amount of public health and medical data held by the National Health Insurance Corporation and the National Health Insurance Review and Assessment Service is approximately 6.4 trillion cases based on the number of insurance claims for medical expenses in South Korea [8], and 92% of the patient data is stored in electronic medical records [9,10]. The government has been initiating several policy measures to increase the use of health care big data. It has been expanding health care big data systems in public institutions, linking big data held by the National Health Insurance Corporation [11] and using it as a single system. Particularly at the national level, the government has introduced the MyData platform to revitalize the use of personal health data. It grants individuals the right to use their health information in a desired way. The Ministry of Health and Welfare has also established a My Healthway platform to support the integration and use of personal medical data. The ministry plans to integrate public health information with medical and personal health information beginning in 2022 [12].

Despite the availability of health big data and pan-government efforts to increase data use, the use of health big data is restricted to public institutions that hold big data and conduct research in public interest [13,14]. Individuals, companies, research institutes, and academia have difficulty accessing and using health big data. This can be attributed to inconsistent data quality, lack of professional help [5], inability to collect and analyze purposeful data, and failure to link and integrate public and private data [15]. In order to increase the use of health big data, it is important to identify and develop measures to meet the various demands for health big data from individuals, private companies, and research institutes, but there is little research in this area. Therefore, this study aims to identify the perceptions and needs of actual users related to the analysis and utilization of health big data and, through this, to identify the main cause of the decline in the utilization of such data. Based on this study, additional studies will be needed to design specific measures that can increase the utilization of health big data in the future.

## Methods

### Research Design

In this study, we conducted a survey to identify the perceptions and demands of health big data analysis and use among workers in health care–related fields.

### Participants

Among the survey panels held by South Korean research, URLs were sent via email and mobile phone to those who met the criteria for this study, and snowball sampling was conducted by sending URLs through social media, mainly to acquaintances of researchers who were engaged in health care–related fields. The specific criteria for selecting participants were as follows: (1) currently working in South Korea as a doctor, nurse, medical technician, pharmacist, hospital employee, pharmaceutical company or medical equipment company employee, health care–related department professor, or health care–related field researcher and (2) those who voluntarily agreed to participate. The number of participants was calculated as 384, which satisfies the confidence level of 95% and the maximum sampling error of 5%, and finally, 400 people were surveyed in consideration of the dropout rate [16-18].

### Questionnaire Items

**Figure 1:**
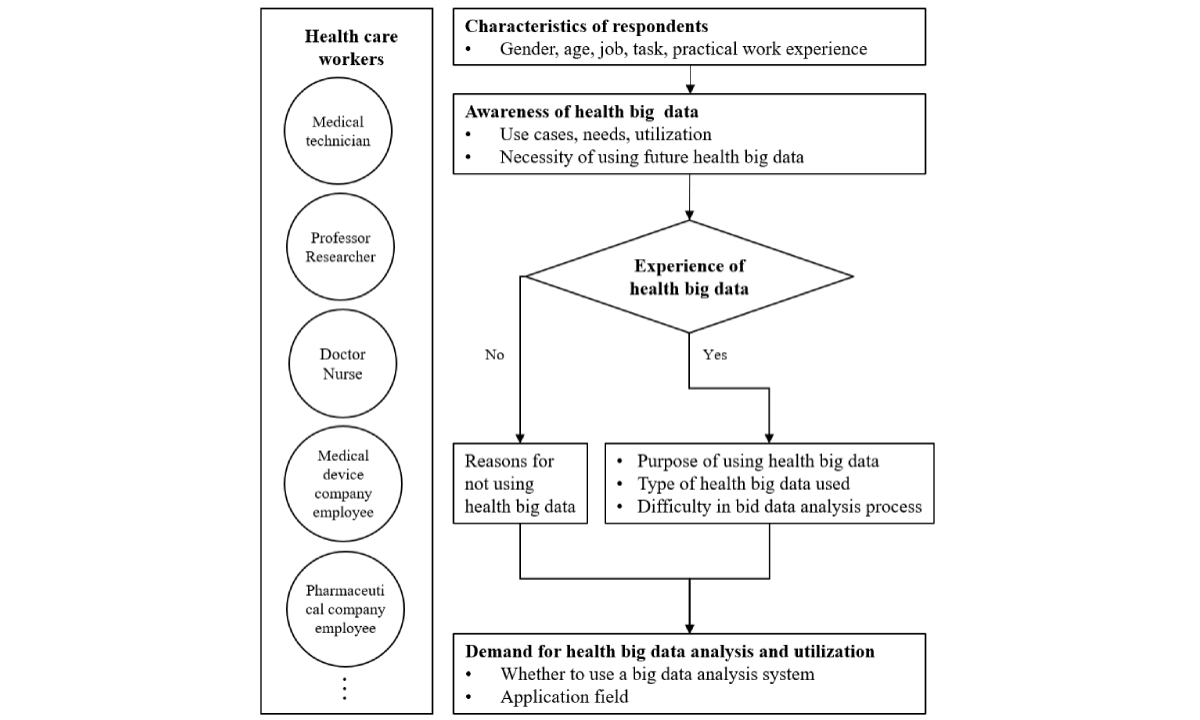
Survey flowchart depicting the analysis and use of health big data among health care workers.

We prepared the questionnaire based on the health insurance big data usage experience and data opening demand survey conducted by the National Health Insurance Corporation [19] and based on the questionnaire used in Baek et al’s [20] study titled “Recognition and Use of Cancer Big Data in South Korea.” To ensure the appropriateness of the questionnaire, we recruited 2 university professors and 2 experts from the health care and information and communication domains to review it. We revised and completed the questionnaire based on the feedback from these reviewers. The 29-question survey was divided into 5 categories, namely, basic information of respondents, awareness and use of health big data, experiences of health big data collection and analysis, demand for health big data analysis and use, and some conditional questions (Figure 1). To gauge respondents’ understanding of health big data, we asked the following questions: “Have you ever witnessed the use of health big data?” and “Have you ever felt the need to use health big data?” In relation to respondents’ current or potential use of health big data, we asked, “Do you have any experience using health big data?” We also asked users whether the situation necessitated the use of health big data. We used the 5-point Likert scale to score the questions on health big data collection and analysis experience. There were questions that measured the degree of difficulty experienced in the process of collecting and analyzing health big data from 1 “very difficult” to 5 “very easy,” meaning that the lower the score, the greater was the difficulty. Finally, in relation to the development of a system to aid in the health big data analysis process, we included a subjective question requesting respondents’ suggestions. To meet the demand for health big data analysis and use, it is necessary to develop a big data system.

### Data Collection

We conducted a web-based survey to collect data from May 8, 2022, to May 18, 2022, after obtaining approval from the bioethics committee of Keimyung University. South Korea Research (a polling company) commissioned the web-based survey. The company sent emails to health care workers in its survey panels. It distributed URLs through emails and simple notification services using the Naver form. We received 400 responses. However, we deleted 10 questionnaires with incomplete responses. This filtration yielded a final sample of 390 questionnaires. We surveyed participants who provided consent to participate after understanding the study’s purpose and their rights to anonymity, confidentiality, and withdrawal. We also gave participants a gift as a token of gratitude for participation.

### Data Analysis

We analyzed data using SPSS 23.0 (IBM Corp). We used Fisher exact test and analysis of variance to determine the differences among occupations. We expressed the results of the analysis by item in frequency and percentage and expressed the difficulties in the health big data analysis process in mean and standard deviation.

### Ethics Approval

This study was approved by the institutional review board of Keimyung University (40525-202112-HR-083-04). The research purpose, methods, and participants’ rights, including that they could cease participation at any point without penalty, were explained. All questionnaires were completed anonymously, and participants were told that the results would not be used for anything other than research purposes. Participation was voluntary, and all respondents provided written informed consent. Those who participated in the web-based survey were given an e-voucher worth US $10.

## Results

### Participants’ General Characteristics

Table 1 shows the demographic characteristics of the respondents. Of the 390 respondents, 178 (45.6%) were males and 212 (54.4%) were females. Regarding age, 76 (19.5%), 141 (36.2%), 119 (30.5%), and 43 (11%) participants were in their 20s, 30s, 40s, and 50s, respectively. With regard to relevant practical work experience, 118 (30.3%), 102 (26.2%), 85 (21.8%), and 85 (21.8%) had experience of 1-2 years, 3-5 years, 6-10 years, and more than 10 years, respectively. Regarding occupations, 34 (8.7%), 47 (2.1%), 79 (20.3%), 24 (6.2%), 55 (14.1%), 44 (11.4%), and 107 (27.4%) respondents were doctors, nurses, medical technicians, hospital administration and computer staff, pharmaceutical and medical company staff, professors and researchers, and personnel from other health care fields, respectively. Regarding respondents’ responsibilities, 156 (40%), 78 (20%), 26 (6.7%), and 12 (3.1%) were working as direct health care providers, administrative and management personnel, marketing personnel, and data processing and information personnel, respectively.

**Table 1:** Baseline characteristics of the respondents (N=390).

Characteristics, categories		Values, n (%)
**Gender**		
	Men	178 (45.6)
	Women	212 (54.4)
**Age (years)**		
	≤29	76 (19.5)
	30-39	141 (36.2)
	40-49	119 (30.5)
	50-59	43 (11)
	≥60	11 (2.8)
**Job**		
	Other health care–related occupations	107 (27.4)
	Medical technician	79 (20.3)
	Nurse	47 (12.1)
	Professor and researcher	44 (11.3)
	Doctor	34 (8.7)
	Pharmaceutical company employee	31 (7.9)
	Medical device company employee	24 (6.2)
	Hospital administration and computer staff	24 (6.2)
**Task**		
	Direct medical care	156 (40)
	Administration and management	78 (20)
	Education and research	62 (15.9)
	Other	29 (7.4)
	Sales and marketing	26 (6.7)
	Product development	16 (4.1)
	Computer and information technology	12 (3.1)
	Service development	11 (2.8)
**Practical work experience (years)**		
	1-2	118 (30.3)
	3-5	102 (26.2)
	6-9	8 (21.8)
	≥10	8 (21.8)

### Awareness of and Experience With Health Big Data

Table 2 shows the perceptions of health big data among the respondents. In total, 47.9% (187/390) and 86.4% (337/390) of the respondents stated that they had witnessed health big data use and that they felt the need to use health big data, respectively. Concerning the current use of health big data, 13.3% (52/390) and 24.9% (97/390) of the respondents stated that they had very low and somewhat low use, respectively, while 38.2% (149/390) of the respondents expressed the need to use health big data in the future, and 65.6% (256/390) and 29.5% (115/390) of the respondents gave full consent and agreed to the future need for big data use, respectively.

**Figure 2:**
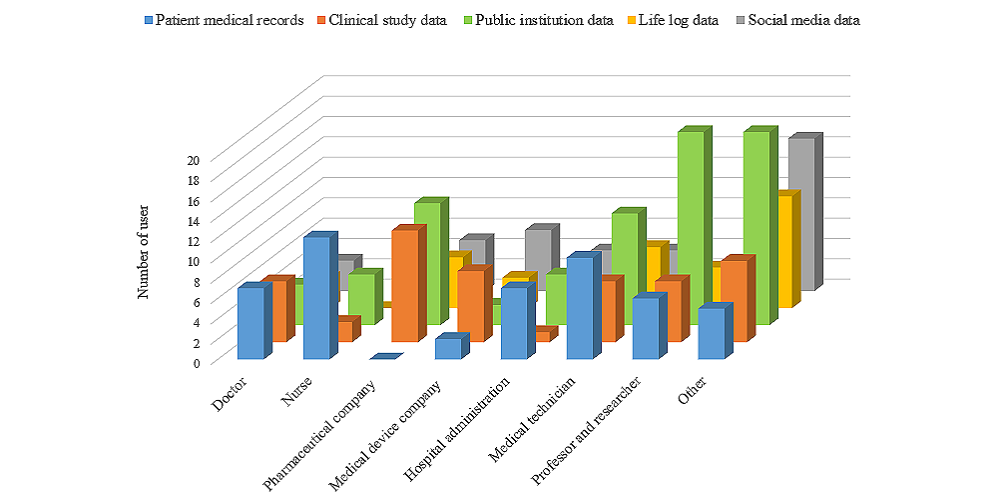
Type of health big data used by health care workers.

Table 3 shows respondents’ use of health big data. Among 65.6% (256/390) of the respondents who never used health big data, 39.6% (153/386) attributed their nonuse to the lack of awareness about the source of the desired data (the most cited reason for nonuse). Among the users, the most cited purpose for using big data was for collecting and utilizing work-related data (62/242, 25.6%), followed by the purposes of predicting and diagnosing diseases (52/242, 21.5%), streamlining other care services (45/242, 18.6%), and academics and research (35/242, 14.5%). Concerning the types of big data used, the most used type of health big data was public institution data (75/249, 30.1%), followed by patient care data (57/249, 22.9%) and clinical study data (44/249, 17.7%). By comparing the types of health big data used by occupation, we found that pharmaceutical or medical companies made substantially less use of patient care data (Figure 2).

**Table 2:** Awareness of health big data (N=390).

Variables		Total (N=390), n (%)	Doctor (n=34), n (%)	Nurse (n=47), n (%)	Pharmaceutical company employee (n=31), n (%)	Medical device company employee (n=24), n (%)	Hospital administrator and computer staff (n=24), n (%)	Other (n=107), n (%)	Medical technician (n=79), n (%)	Professor or researcher (n=44), n (%)	Fisher exact *P* value	
**Have you ever seen examples of health big data being used in the health care field?**												<.001
	Yes	187 (47.9)	15 (44.1)	30 (63.8)	21 (67.7)	18 (75)	14 (58.3)	34 (31.8)	25 (56.8)	30 (38)		
	No	203 (52.1)	19 (55.9)	17 (36.2)	10 (32.3)	6 (25)	10 (41.7)	73 (68.2)	19 (43.2)	49 (62)		
**Have you ever felt the need to use health big data in a work-related field?**												.01
	Yes	337 (86.4)	24 (70.6)	42 (59.4)	25 (80.6)	19 (79.2)	24 (100)	101 (94.4)	42 (95.5)	60 (75.9)		
	No	53 (13.6)	10 (29.4)	5 (10.6)	6 (19.4)	5 (20.8)	0 (0)	6 (5.6)	2 (4.5)	19 (24.1)		
**Do you think health big data are being used well in the current health care field?**												.66
	Not at all	52 (13.3)	2 (5.9)	4 (8.5)	1 (3.2)	3 (12.5)	3 (12.5)	21 (19.6)	10 (12.7)	8 (18.2)		
	Not very well	97 (24.9)	6 (17.6)	11 (23.4)	4 (12.9)	6 (25)	1 (4.2)	35 (32.7)	26 (32.9)	8 (18.2)		
	Usually	149 (38.2)	20 (58.8)	21 (44.7)	16 (51.6)	6 (25)	14 (58.3)	28 (26.2)	33 (41.8)	11 (25)		
	Well	65 (16.7)	4 (11.8)	10 (21.3)	7 (22.6)	6 (25)	4 (16.7)	18 (16.8)	7 (8.9)	9 (20.5)		
	Very well	27 (6.9)	2 (5.9)	1 (2.1)	3 (9.7)	3 (12.5)	2 (8.3)	5 (4.7)	3 (3.8)	8 (18.2)		
**Do you think you need to use big data in additional health care fields?**												.88
	Strongly agree	256 (65.6)	14 (41.2)	34 (72.3)	25 (80.6)	16 (66.7)	12 (50)	76 (71)	41 (51.9)	38 (86.4)		
	Agree	115 (29.5)	13 (38.2)	10 (21.3)	4 (12.9)	6 (25)	12 (50)	29 (27.1)	35 (44.3)	6 (13.6)		
	Disagree	7 (1.8)	3 (8.8)	1 (2.1)	1 (3.2)	0 (0)	0 (0)	1 (0.9)	1 (1.3)	0 (0)		
	Strongly disagree	3 (0.8)	1 (2.9)	1 (2.1)	0 (0)	0 (0)	0 (0)	0 (0)	1 (1.3)	0 (0)		
	Unknown	9 (2.3)	3 (8.8)	1 (2.1)	1 (3.2)	2 (8.3)	1 (0.9)	0 (0)	1 (1.3)	0 (0)		

**Table 3:** Experience using health big data.

Usage of health big data			Values, n (%)
**Do you have any experience using health big data? (N=390)**			
	Yes	134 (34.4)	
	No	256 (65.6)	
**Why have you not used health big data? (multiple responses) (n=386)^a^**			
	Difficulty selecting and organizing data suitable for the purpose	85 (22)	
	I don’t know where to collect the necessary data (difficulty with data access)	153 (39.6)	
	Difficulty in the selection of an analysis method and data analysis	102 (26.4)	
	No need for data	42 (10.9)	
	Other	4 (1)	
**What is your purpose in using health big data? (multiple responses) (n=242)^b^**			
	Collection and use of work-related data	62 (25.6)	
	Prediction and diagnosis of disease	52 (21.5)	
	Efficiency of medical services	45 (18.6)	
	Research	35 (14.5)	
	Health care product development	26 (10.7)	
	Health care service development	22 (9.1)	
**What type of health big data have you used? (multiple responses) (n=249)^c^**			
	Public institution data (health checkup result, statistical data)	75 (30.1)	
	Patient medical records (electronic medical record, radiographic image, prescription)	57 (22.9)	
	Clinical study data (clinical trial, genetic investigation, new drug test)	44 (17.7)	
	Social media data (health-related data collected from social network service and portal)	39 (15.7)	
	Life log data (wearables, health care app data, home monitoring, Internet of Things)	34 (13.7)	

^a^This question confirmed the reason that big data could not be used by 256 participants who had no experience in using big data in a multiresponse manner, and 386 responses were obtained.

^b^This question confirmed the purpose of using big data in a multiresponse manner by 134 participants with experience in using big data, and 242 responses were obtained.

^c^This question identified the type of big data used by 134 participants who had experience using big data, and only 249 responses were obtained.

### Experience With Health Big Data Collection and Analysis

Table 4 shows respondents’ experiences with health big data collection and analysis. Experience “integrating different data and matching expression units” received the lowest score of 2.39 points out of 5 points. Experience “processing missing values and removing noise from collected data” and experience “removing and converting data into easily understandable formats” scored 2.50 points and 2.61 points, respectively. Experience “converting collected data into structural forms and storing and managing data” and experience “choosing appropriate analysis techniques” scored low at 2.71 points and 2.78 points, respectively. These findings confirmed that participants faced difficulties in data preconditioning during the health big data analysis process.

**Figure 3:**
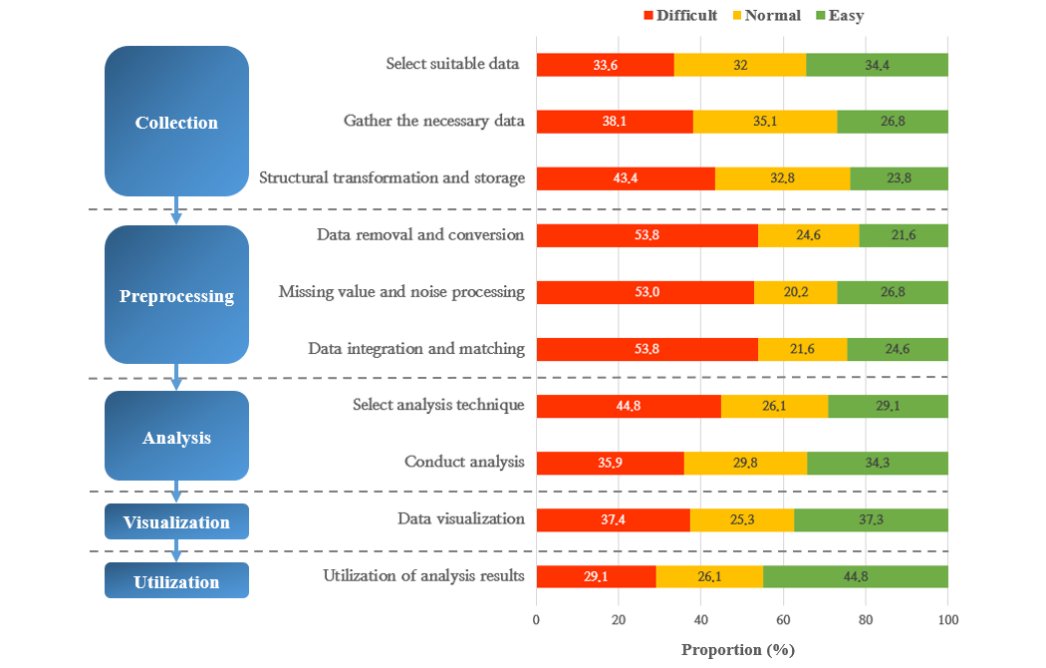
Obstacles to health big data use among health care workers.

We compared the difficulties faced by different professionals during the big data analysis process. Professionals from 5 health care fields had the lowest score during the process. Among them, doctors had difficulties integrating different data and matching units of expression. This finding confirmed that participants experienced common difficulties, regardless of occupation. Among all the professionals, nurses and pharmaceutical company employees had the lowest scores (Figure 3).

**Table 4:** Experience with health big data collection and analysis (n=134).

Ease of using the health big data by procedure			Values, mean (SD)
**Data collection**			
	Selection and composition of data suitable for the purpose	2.99 (1.04)	
	Appropriate data collection	2.80 (0.96)	
	Conversion and storage in the structured form of the collected data	2.71 (1.05)	
**Data preprocessing**			
	Removal of unsuitable data and type conversion into a form that is easy to analyze	2.61 (1.03)	
	Missing value handling and noise removal	2.50 (1.04)	
	Integration of data from different sources and matching of units of expression	2.39 (1.04)	
**Data analysis**			
	Choosing an appropriate analytical technique	2.78 (1.05)	
	Analysis	2.87 (0.97)	
**Data visualization**			
	Visualization of analysis results	2.85 (1.03)	
**Data utilization**			
	Applied in practice	2.94 (0.88)	

### Demand of Health Big Data Analysis and Utilization

Table 5 shows respondents’ demand for the analysis and use of health big data, with 91.8% (358/390) expressing the need for a system that can facilitate big data analysis and use. This need followed the need to develop health care services. Concerning the application of health big data, 21.1% (214/1016), 12.5% (127/1016), 14.8% (150/1016), 13.2% (134/1016), 12.5% (127/1016), and 12.3% (125/1016) of the respondents stated that health big data should be used for health care service development, individual disease prediction, chronic disease prediction, working process improvement, health care product development, and disease surveillance system development in communities and companies, respectively.

**Table 5:** Demand for health big data analysis and utilization.

Variables			Values, n (%)
**If there is a system that supports a series of processes from big data collection to utilization, would you like to use it? (N=390)**			
	Yes	358 (91.8)	
	No	32 (8.2)	
**What do you want to do with health big data? (multiple responses), (n=1016)**			
	Health care service development	214 (21.1)	
	Prediction of individual disease occurrence	185 (18.2)	
	Prediction of chronic diseases	150 (14.8)	
	Improvement of work process	134 (13.2)	
	Health care product development	127 (12.5)	
	Disease monitoring system for workplace or region	125 (12.3)	
	Evaluation of the appropriateness of medical expenses	80 (7.9)	
	Other	1 (0.3)	

### Open-ended Questions

We received 210 answers to the subjective questions regarding the development of a system that helps analyze big data. The respondents who highlighted the need to improve access to personal health and medical care data and to develop a system to support data collection of these data accounted for 18.1% (38/210). This was followed by the need for professional guidance on the selection and configuration of appropriate data (26/210, 12%), providing advice on questions arising during the overall process (34/210, 16.1%) and easier data analysis module development (23/210, 10.9%). Other recommendations included the need to compare analytical techniques and provide guidance on appropriate analytical techniques (18/210, 8.5%); increase the ease of use and access (18/210, 8.5%); and provide training and guidance manuals for program use (15/210, 7.1%), guidance (10/210, 4.7%), and simple procedure data integration systems (8/210, 3.8%). In relation to increasing the usability of health big data, respondents highlighted the need to provide education on and promote such data (17/210, 8.1%). This was followed by opinions on the need to consider views on developing measures for protecting personal information (10/210, 7.5%). Few respondents also provided opinions on the need to develop a data classification system, provide expert feedback on the interpretation of analyzed data, and revise law for convenient procedures.

## Discussion

### Principal Findings

This study was conducted to understand the perception of and demand for health big data analysis and use among domestic health care–related workers in South Korea. According to our survey of 390 health care–related workers, 86.4% (337/390) of the respondents felt the need to use health big data and 95.1% (371/390) recognized the need to use big data in the future. However, only 23.6% (92/390) of the respondents stated that health big data are being satisfactorily used and that it is yet to reach its full potential. Only 47.9% (187/390) of the respondents had seen the use of health big data, which reflects the low awareness and knowledge of health big data use among health care professionals. Among the 65.6% (256/390) of the respondents who had never used health big data, 39.6% (153/386) attributed their nonuse to the lack of awareness about the source of the desired data (the most cited reason for nonuse).

Despite various attempts to increase health big data use, such as government-level efforts, legal reforms, and the establishment of health big data systems by public institutions, individuals still have difficulty accessing health big data. Currently, a service that guides the introduction and access procedure of public big data that are also available on the government-built health care big data platform is provided. However, even this can be considered difficult for beginners with no analysis experience; thus, a system that enables immediate question-and-answer with a more specific explanation should be combined. Many of those who have experience using health big data have experienced significant difficulties in tasks such as “defects in other data sources” and “missing value processing and noise removal,” which are part of the data preprocessing stage during the big data analysis process. However, these data integration and purification processes are closely related to the quality of the data. As the quality of data plays a significant role in the accuracy of the analysis results, it is important to solve these issues [21]. Although patient treatment data were highly utilized in hospitals by doctors, nurses, and administrative and computer workers, their use was low in other industries such as pharmaceutical and medical instrument companies. These findings imply that nonhospital workers may find it challenging to use health big data, as clinical data are difficult to access and use due to issues of data standardization (ie, data structure and governance is unique to each hospital). Hence, it is urgent to develop a platform to expand accessibility and ease of use by standardizing and constructing hospital data through systems such as the electronic medical records certification system. The government is currently promoting the development of the Common Data Model [22].

In this study, many respondents wanted to use the health big data actively to develop useful programs or products that contribute to health care and promotion in the future. To date, health big data use has primarily been limited to the collection or utilization of work-related data or the purpose of academic research. In addition to the innovative development of digital technology after COVID-19, various digital health care services are rapidly emerging in the health and medical field [23,24]. If accurate predictions and evidence-based judgments based on health big data are applied to the development of health care services or products developed in various health-related fields such as diet, beauty, and exercise, more precise services can be provided to users [25,26]. In the case of health care fields, professionals can develop prediction models via data mining technologies. This can help them analyze large data sets of patient data and identify different clusters from and correlations between data sets [27]. The use of health big data with artificial intelligence technologies such as machine learning and deep learning can further facilitate innovation in health care services, such as individual clinical decision support systems and real-time precision diagnosis [28,29]. Organizations can use big data based on artificial intelligence to address cost and efficiency problems emerging during drug trials [9]. Therefore, supporting the use of health big data for various purposes in various fields can lead to a paradigm shift in the health care field.

In this study, one of the main factors hindering the use of health big data was the difficulty in the analysis process, and most of the respondents expressed wanting a support system to help with the big data analysis process. In particular, many respondents noted the need for services that provide expert guidance or advice in the overall process of data collection, preprocessing, analysis, and interpretation of analysis results. However, it is not easy to establish a support system that provides real-time communication with experts in the current situation, wherein there is a substantial shortage of experts in big data analysis. Therefore, it is expected that users’ difficulty can be largely addressed if an interactive system is developed to enable them to communicate with real-time users on behalf of such expert roles. In addition, with the increasing demand for non–face-to-face services in the post–COVID-19 era, an intact system can be more useful [30]. However, users’ level of experience with big data analysis varies widely from beginners to experts; thus, the developed support system should be designed to meet the level and needs of users [31]. Further, more specialized support should be provided for the specific challenges that arise at each stage during the entire process of big data analysis [32]. Recently, the scope of the user-centered design methodology in industrial design has been expanded and applied not only to the development of products with external shapes but also to the development of technologies or services [33]. This approach enables customized design by specifically identifying user requirements and problem situations and reflecting them in the service design [34]. Therefore, in developing a support system that helps analyze and utilize health big data, reflecting the empirical characteristics of the participant should be considered first.

Currently, various national policies and projects are being promoted for the effective use of health big data based on large public health data holdings and advanced digital technology, and as a representative example, a health care big data platform has been developed and provided. However, these achievements developed with substantial financial input have not led to the active use of health big data in the actual health care field. Therefore, to ensure that new health care data–related projects that are currently or continuously promoted in the future do not merely remain as tangible results, it is urgent to specifically identify and resolve the barriers to entry to health big data for end users. Based on this study, we propose a study on the development of customized health big data analysis and utilization support services tailored to users’ needs, and ahead of that, a follow-up study is needed to identify the specific needs of health big data users.

### Limitations

This study has several limitations. First, to check the answers of the participants related to health big data collection and analysis, we selected some participants through snowball sampling, starting with those who had analyzed health big data; however, this is not a probability sample for health care workers, and the investigation is limited to the situation in South Korea. Second, as the questionnaire has not been verified for objective reliability and validity, there may be limitations in interpreting the results. Third, this study was conducted over a relatively short period of time, and the contents confirmed in the study are the general experiences and requirements of various workers in the health care field; therefore, it is difficult to identify specific needs subdivided by the participants. In the future, a follow-up study is needed to subdivide the participants and divide them by type to confirm participants’ in-depth experience by type.

### Conclusions

In this study, respondents strongly perceived the need for health big data. However, the actual use was low in the related field. They attributed the reasons for low utilization to difficulty getting access to data and difficulty in the analysis process. They highlighted the need to develop a system that can assist them at each stage of the data analysis process. This is expected to increase big data use. Therefore, this study attempts to identify the main issues hindering the use of health big data by investigating the perceptions of the health big data and the experiences and needs related to big data analysis among various health care workers. This study is meaningful as the first attempt to identify critical failure factors that are easily overlooked in health big data utilization, and it is hoped that various attempts and studies will continue to discover and solve more specific and detailed utilization problems in the health big data area in the future.

## Data Availability

The data sets generated or analyzed during this study are available from the corresponding author on reasonable request.

## References

[1] Lee J (2020). The big data policy in the data economy. https://innovation.jams.or.kr/co/com/EgovMenu.kci?s_url=/ac/conference/main/jmMain.kci&s_MenuId=MENU-000000000030000&accnId=AC0000000002%20.

[2] Newman D How to plan, participate and prosper in the data economy. Gartner.

[3] Dash S, Shakyawar S, Sharma M, Kaushik S (2019). Big data in healthcare: management, analysis and future prospects. J Big Data.

[4] Lemoine C (2014). Precision medicine for nurses: 101. Semin Oncol Nurs.

[5] Kwon S (2021). Utilization Assignment of Medical Information Healthcare in The 4th Industrial Revolution Era. JKCI.

[6] Pastorino R, De Vito Corrado, Migliara G, Glocker K, Binenbaum I, Ricciardi W, Boccia S (2019). Benefits and challenges of Big Data in healthcare: an overview of the European initiatives. Eur J Public Health.

[7] Batko K, Ślęzak A (2022). The use of Big Data Analytics in healthcare. J Big Data.

[8] Moon J (2021). Activation of health care big data. J Korea Inst Inf Commun Eng.

[9] Bae B Discovering opportunities for new drug development and patient treatment: How medical real-data can be used. HIRA Res.

[10] Health care data utilization innovation. Korea Health Information Service.

[11] Choi J, Nam T, Cho M (2020). Issues related to the public use of health care big data and medical platform: Focusing on the implementation of the health care big data. Journal of Governance Studies.

[12] Lee K (2021). Current status of MyData policy and tasks in health and welfare. Health Welf Policy Forum.

[13] Lee Y (2015). Big data and its applications in the health and welfare sectors. Health Welf Policy Forum.

[14] Lim G (2020). Health and welfare data utilization and challenges. Health Welf Policy Forum.

[15] Shin S (2018). Standardize unstructured health care data. The J Korean Inst Commun Inf Sci.

[16] Swanston E, Pulman A, Dogan H, Murphy J, Bitters F (2021). Scoping the Need for a Tailored mHealth App to Improve Health and Well-being Behavioral Transformation in the Police: Exploring the Views of UK Police Workers via Web-Based Surveys and Client Meetings. JMIR Form Res.

[17] Kim H (2019). A study on parking user's perception for vitalizing the shared parking in residential priority parking areas. KSCE Journal of Civil and Environmental Engineering Research.

[18] Tesfa GA, Yehualashet DE, Ewune HA, Zemeskel AG, Kalayou MH, Seboka BT (2022). eHealth Literacy and its Associated Factors Among Health Professionals During the COVID-19 Pandemic in Resource-Limited Settings: Cross-sectional Study. JMIR Form Res.

[19] Health insurance corporation begins preparations for data openness experienced by the public. National Health Insurance.

[20] Baek ES, Lee C, Chang JS, Choi JE, Shin SJ (2021). The Awareness and Usage of Big Data for Cancer in Korea: A Survey Study. J Health Info Stat.

[21] Ambigavathi M (2018). A survey on big data in health care applications. Intelligent Communication, Control and Devices.

[22] Lee C, Kim J, Noh S, Kim T, Lee Y, Yu Y (2020). Medical big data-based extended artificial intelligence integration platform. Korea Inform Process Society.

[23] Kwon H, Kim Ho Heon, An Jaeil, Lee Jae-Ho, Park Yu Rang (2021). Lifelog Data-Based Prediction Model of Digital Health Care App Customer Churn: Retrospective Observational Study. J Med Internet Res.

[24] Han J, Kim EJ (2020). Smart health care. KISTEP Technology Trend Brief.

[25] Shin KS (2021). Smart health care technology accelerating with smart cities. The Transactions of the Korean Institute of Power Electronics.

[26] Hulsen T, Jamuar S, Moody A, Karnes J, Varga O, Hedensted S, Spreafico R, Hafler D, McKinney E (2019). From Big Data to Precision Medicine. Front Med (Lausanne).

[27] Viceconti M, Hunter P, Hose R (2015). Big Data, Big Knowledge: Big Data for Personalized Healthcare. IEEE J. Biomed. Health Inform.

[28] Wang Y, Hajli N (2017). Exploring the path to big data analytics success in healthcare. Journal of Business Research.

[29] Kim G, Kim H (2021). A Research of Big Data Generation and Utilization Model in Healthcare Field in the Post-Corona Era. HSS21.

[30] Oh M (2019). Strategies and tasks for big data in health and welfare. Health Welf Policy Forum.

[31] Lee M, Lee S, Lee W, Koo K, Han J, Yoo J (2021). An investigation of user demand from a perspective of business modelling for developing a need-based smart design platform.

[32] Veryzer R, Borja de Mozota B (2005). The Impact of User-Oriented Design on New Product Development: An Examination of Fundamental Relationships*. Journal of Product Innovation Management.

[33] Alberts NM, Badawy SM, Hodges J, Estepp JH, Nwosu C, Khan H, Smeltzer MP, Homayouni R, Norell S, Klesges L, Porter JS, Hankins JS (2020). Development of the InCharge Health Mobile App to Improve Adherence to Hydroxyurea in Patients With Sickle Cell Disease: User-Centered Design Approach. JMIR Mhealth Uhealth.

[34] User centered design. Interaction Design Foundation.

